# Supplementary material for: Gintonin Alleviates HCl/Ethanol- and Indomethacin-Induced Gastric Ulcers in Mice
Source: Int J Mol Sci. 2023 Nov 24;24(23):16721. doi: 10.3390/ijms242316721 (PMC10705886; doi:10.3390/ijms242316721)
Supplement: Supplementary file 1 [file ijms-24-16721-s001.zip › ijms-2704626-supplementary.pdf]

Supplementary Data

Gintonin alleviates HCl/ethanol- and indomethacin-induced gastric ulcer in mice

Han-Sung Cho [1,a], Tae Woo Kwon [2,a], Ji-Hun Kim [1], Rami Lee [1], Chun-Sik Bae [3], Hyoung-Chun Kim [4], Jong-Hoon Kim [5], Sun-Hye Choi [6], Ik-Hyun Cho [2,*], and Seung-Yeol Nah [1,*]

[1] Ginsentology Research Laboratory and Department of Physiology, College of Veterinary Medicine, Konkuk University, Seoul 05029, Korea
[2] Department of Convergence Medical Science, College of Korean Medicine, Kyung Hee University, Seoul, 02447, Republic of Korea
[3] College of Veterinary Medicine, Chonnam National University, Gwangju, Republic of Korea
[4] Neuropsychopharmacology and Toxicology Program, College of Pharmacy, Kangwon National University, Chunchon, Republic of Korea
[5] College of Veterinary Medicine, Biosafety Research Institute, Chonbuk National University, Iksan-city, Jeollabuk-Do, Republic of Korea
[6] Department of Animal Health, College of Health and Medical Services, Osan University, Osan-si, Republic of Korea
[a] Han-Sung Cho and Tae Woo Kwon equally contributed to this work.
* Correspondence: ihcho@khu.ac.kr (I.-H.C.); synah@konkuk.ac.kr (S.-Y.N.);
Tel.: + 82-2-450-4154; Fax: +82-2-450-3037 (S.-Y.N.)

Materials and Methods

Western Blot Analysis

The mice were anesthetized with urethane and cardiac perfusion was performed using 1xPBS after astric ulcer was induction with 0.3 M HCl/ethanol or indomethacin. The excised gastric tissues were homogenized in the 2 ml tube and then centrifuged at 13000 rpm and 4°C for 20 min to obtain a precipitate. The supernatant was removed after centrifugation and 100 μl of Radioimmunoprecipitation assay (RIPA) lysis buffer (150 mM Sodium Chloride, 0.25% Sodium deoxy-cholate, 1 mM EGTA, 1% NP-40, 50 mM Tris-HCl pH 8.0) was added to the precipitate, resting on ice for 1 h. After centrifugation at 13000 rpm and 4°C for 20 min, the supernatant was subjected to protein quantification using the BCA protein kit (Thermo-Fisher Scientific Korea, Gangnam-gu, Seoul, Korea). All the samples were analyzed after protein quantification. Samples were further processed according to Lee et al (2017) [44] using LPA2R antibody (1:1000, Cat. No. ab38322, abcam, Cambridge, UK).

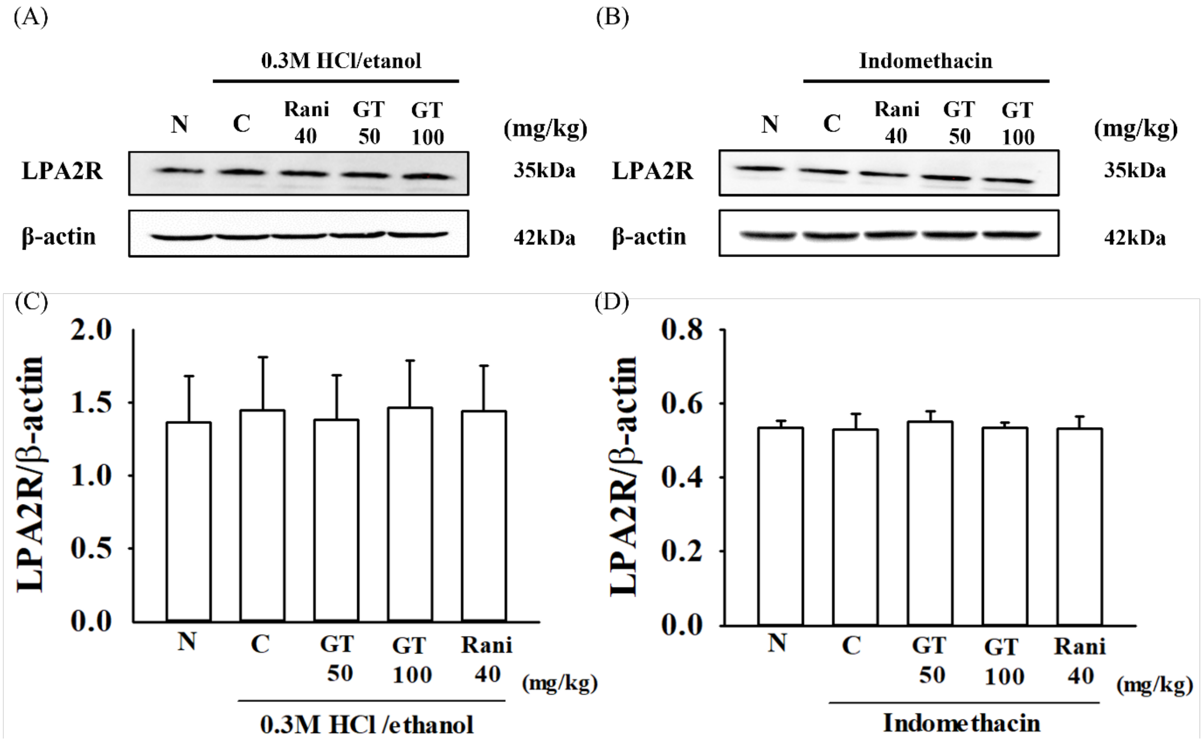

Supplementary Figure S1. Lysophosphatidic acid (LPA)2 receptor expression after gintonin treatment in gastric ulcer-induced mice. LPA2 receptor (LPA2R) protein expression was detected by western blot analysis in gastric ulcer-induced mice (A) After mice were treated by gintonin (either 50 mg/kg (GT 50) or 100 mg/kg (GT 100)), or ranitidine (40 mg/kg) for 1 hour individually, 0.3M HCl/ethanol was treated to mice for another 1 h. (B) Mice were treated with gintonin (either 50 mg/kg (GT 50) or 100 mg/kg (GT 100)), or ranitidine (40 mg/kg) for 1 hour individually and then treated with indomethacin for another 12 h. (C and D). The summarized histogram was shown as LPA2R/β-actin ratio at each dose. The data are expressed as the mean ± standard error of mean (n=5). There was no significant difference between GT group and normal/or control group.
